# Relationship between musculoskeletal pain, sleep quality and migraine with level of physical activity in college students during the COVID-19 pandemic

**DOI:** 10.1016/j.heliyon.2022.e10821

**Published:** 2022-09-29

**Authors:** Juliana Pedrosa Luna Oliveira, Juliana Zangirolami-Raimundo, Paulo Evaristo de Andrade, Soraya Louise Pereira Lima, Amanda Regina Cavalcante Lima, Luiz Carlos de Abreu, Rodrigo Daminello Raimundo

**Affiliations:** a Department of Physiotherapy, Faculty of Integration of the Sertão, Serra Talhada, Pernambuco, Brazil; b Gynecology Discipline, Department of Obstetrics and Gynecology, Faculty of Medicine FMUSP, University of São Paulo, São Paulo, SP, Brazil; c Laboratory of Study Design and Scientific Writing, Faculdade de Medicina da ABC, Santo André, SP, Brazil; d School of Medicine, University of Limerick, Castletroy, Co. Limerick, Ireland

**Keywords:** COVID-19, Musculoskeletal pain, Migraine, Sleep wake disorders, Exercise

## Abstract

**Background:**

The COVID-19 pandemic has negative impacts on general health of the population, social isolation can contribute to the emergence of various dysfunctions.

**Objective:**

To investigate the association musculoskeletal pain, sleep quality and migraine with the level of physical activity during the COVID-19 pandemic in college students.

**Method:**

Data were collected through a sociodemographic questionnaire containing questions regarding sample characterization, the Nordic Musculoskeletal Questionnaire (NMQ), Pittsburgh Sleep Quality Index (PSQI), Migraine Disability Assessment (MIDAS) and the International Physical Activity Questionnaire (IPAQ).

**Results:**

In the correlation made between the data at the beginning of the pandemic, there was a direct relationship between PSQI and the number of days with pain (p < 0.001), the Initial MIDAS score (p < 0.001) and the initial pain intensity (p < 0.001). There was a direct relationship between PSQI scores and age (p = 0.044), MIDAS (p < 0.001) and pain intensity (p < 0.001). We identified a direct relationship between MIDAS and the number of days with pain (p < 0.001) and pain intensity (p < 0.001).

**Conclusion:**

Social isolation, during the COVID-19 pandemic, probably potentiated painful symptoms in various parts of the body, worsening sleep quality and migraine. In addition, there is a strong evidence that the decrease in physical activity during the pandemic is associated with sleep quality, with the number of days with musculoskeletal pain and migraine.

## 1 Introduction

In December 2019, a new coronavirus causing SARS-COV-2 (COVID-19) emerged in China, which is an infectious disease caused by an RNA virus, that is a simple-tape virus and therefore presents greater speed in the generation of new copies of viruses, presenting stronger infection capacity and several transmission channels, besides being associated with the high mortality rate of affected people, leading the World Health Organization (WHO) to declare on March 11, 2020 the state of pandemic [1].

Given the state of pandemic, several countries in the world, including Brazil, have enacted lockdown, that is, a protocol of isolation or confinement reducing and/or preventing the movement of people in order to reduce the transmission of the virus. In this scenario, schools and universities stopped classroom classes, leading the entire sector to review teaching-learning strategies. Remote teaching, with online classes, was the main strategy adopted by Higher Education Institutions (HEIs) to continue the programcontent of the period/school year [2].

Due to the troubled period we are going through and the attitudes adopted by students during the performance of their academic activities in their homes, keeping the neck flexed when using portable devices, and constant and repetitive movements has been “the most likely explanation” for the significant increase in the prevalence of neck-shoulder pain among the population aged 20–34 years in recent decades, becoming vulnerable to the development of severe musculoskeletal injuries, whose symptoms may include: fatigue and pain in the arms, shoulders, neck, and hands and fingers [3, 4, 5].

Associated with musculoskeletal pain, insomnia is characterized as a subjective perception and dissatisfaction with the quality and/or amount of sleep, with difficulty falling asleep, keeping it waking up many times at night or even waking up early, and the individual has a non-restorative sleep. Still on non-restorative sleep, it is known that it has a great impact on work activities and socially speaking, negatively interfering in quality life of the individual, promoting decreased energy, concentration, attention and increased fatigue and daytime malaise [6, 7].

In addition to all disorders caused by decreased sleep quality, migraine can be a consequence of insomnia and negatively affects the quality life of patients, causing functional disability at work and in social life due to associated symptoms [8, 9].

All disorders mentioned above can be mitigated through the practice of physical activity and the fight against sedentary lifestyle, because physical exercises are an important component of healthy lifestyle and bring several health benefits, on the other hand, there are no studies associating the COVID-19 pandemic with these disorders [10, 11].

In view of this, the negative impact of the pandemic on our general health is beginning to emerge. Assuming that the pandemic can contribute to the emergence of musculoskeletal injuries in college students, affect sleep quality due to disturbance and shortened duration; in addition to episodes of headache and/or migraine and that research involving these dysfunctions during the COVID-19 pandemic are scarce, makes studies of this large duration important. Since they can be a powerful tool for the implementation of strategies aimed at minimizing these physical and psychosomatic disorders and preparing health professionals for the care of this type of patient, besides being an innovative work in the area.

In this connection, is it possible to say that the pandemic had a negative influence on the practice of physical activity and that this had consequences for the emergence of musculoskeletal pain in college students, affected quality of sleep, in addition to promoting migraine episodes? Our hypothesis is that there was a relationship between decreased physical activity and musculoskeletal pain as well as sleep quality and migraine.

Therefore, so the objective of this study was to correlate musculoskeletal pain using the Nordic Musculoskeletal Symptom Questionnaire (NMQ), sleep quality through the Pittsburg Sleep Quality Index (PSQI) and the migraine level assessed through the Migraine Disability Assessment (MIDAS), with the level of physical activity through the International Physical Activity Questionnaire (IPAQ), commonly used to study the association of physical activity with health outcomes, in college students during the COVID-19 pandemic.

The paper is arranged as follows. The next section presents the “method” with sub-sections like, study design, population, data collection, ethical aspects and statistical analysis. The subsequent section details the Results. The discussion of findings is provided in the “discussion” section. Conclusions and suggestions are presented in the final section.

## 2 Method

### 2.1 Study design and population

**Figure 1:**
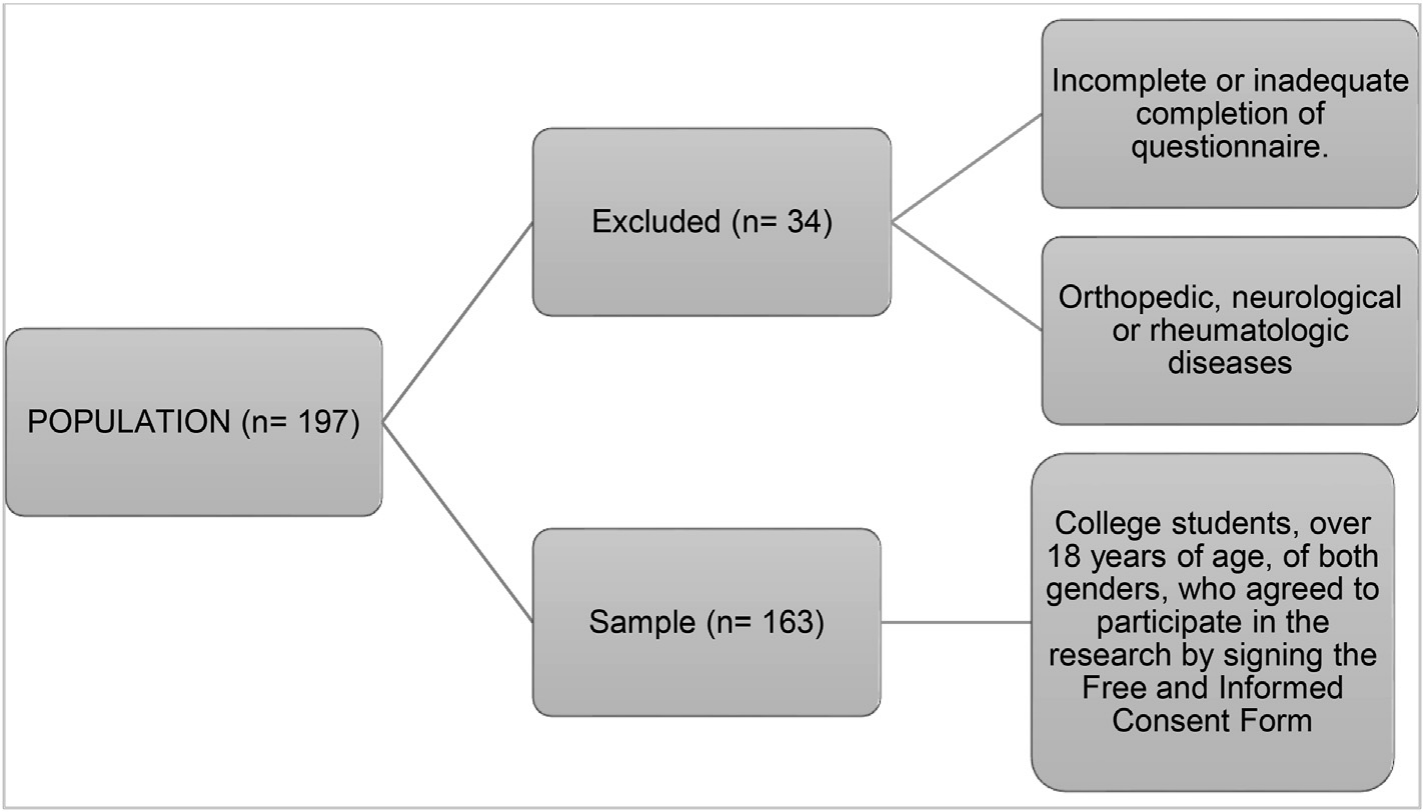
Flowchart of the population studied.

The sample consisted of undergraduate students, of both sexes, regularly enrolled in a Higher Education Institution (HEIs), who were at least 18 years old, without associated psychic pathologies, who agreed to participate in the research voluntarily and who signed the Free and Informed Consent Form. Individuals who performed incomplete or inadequate completion of the questionnaires, history of intervention or orthopedic and neurological diseases, rheumatologic diseases, sensory deficit, and neck trauma during the last six months were excluded from the study (Figure 1).

### 2.2 Procedure

Data were collected online through the Google Forms platform at two different moments. Initially, immediately after the government’s decree of social isolation in March 2020 and the second collection was carried out three months after the isolation, in June 2020.

The main researcher contacted the course coordinators of the educational institutions, explaining about the research and requesting access to the students' instant messaging application groups so that students could be recruited to participate, after accessing the groups links to the survey were sent. To minimize selection bias, some strategies were used: students who did not respond were again invoked privately and another attempt was made to contact the research subjects; in addition, all procedures were standardized by a single researcher. As the choice of messaging application groups was made by the coordination of the courses, the researchers could not define who would be part of the sample, further reducing the selection bias. Before having access to the questionnaires, the students read the Informed Consent Form and signed agreeing to participate in the election. After agreeing, the participants answered the questionnaires used in the research and described below.

### 2.3 Data collection

#### 2.3.1 Socio-demographic questionnaire on social isolation during the COVID-19 pandemic

Sociodemographic indicators were evaluated and data from the following independent variables were collected: sex, age, marital status, city, course and period, previous illnesses, headache and information about social isolation.

#### 2.3.2 Nordic Musculoskeletal Questionnaire (NMQ)

The NMQ consists of an image of the human body, divided into nine body areas in which the participant should record the frequency in which he/she has felt pain, numbness, tingle or discomfort in the regions numbered in the body design through an instrument of multiple choices, on a scale from zero to four, in which 0 represents not feeling discomfort, 1 reports feeling rarely, 2 reports feeling frequently and 3 feels pain or discomfort always and related to the annual and weekly prevalence, validated by [12].

#### 2.3.3 Pittsburgh Sleep Quality Index (PSQI)

The Pittsburgh Sleep Quality Index (PSQI) assesses total sleep time, self-reported sleep quality, and disorders present over the past month. It contains 19 questions distributed in 7 domains. Each domain is ranked on a scale from 0 (zero) to 3 (three) points. The greater the sum of the values, the worse the sleep quality. We used a cutoff value of 5 or more in the final score (sum of components) to indicate reduction in sleep quality [9, 13].

#### 2.3.4 Migraine disability assessment (MIDAS)

MIDAS assesses how headache affects the lives of participants in the last 90 days. The questionnaire consists of seven items [14].

According to these issues, patients should indicate the number of days when they were unable to go to school/work, perform housework and/or have decreased efficiency to perform activities of daily living. At the end, the sum of the number of days is made and the score is evaluated [15].

#### 2.3.5 International Physical Activity Questionnaire (IPAQ)

The IPAQ (International Physical Activity Questionnaire) – Long Version, is composed of twenty-seven questions related to physical activities performed in a normal week, estimating the time spent in activities of vigorous intensity, moderate and mild, with a minimum duration of 10 continuous minutes, in different contexts of daily life [10].

### 2.4 Ethical aspects

Because it is research involving human beings, the researcher undertook to comply with the legal ethical aspects in accordance with Resolutions No. 466/12 and No. 510/2016 of the National Health Council, which provides for guidelines and regulatory standards for research in human beings. The research was previously approved by the Ethics and Research Committee on Human Beings of the Faculty of Integration of the Sertão – FIS (number 3662368).

### 2.5 Statistical analysis

**Figure 2:**
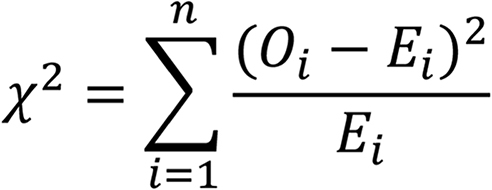
Chi-square test equation ($\chi^{2}$ = Pearson's Cumulative Test Statistics, approximates the chi-square distribution; $O_{i}$ = Observed values; $E_{i}$= Expected values - theoretical; *n* = The number of cells in the table).

**Figure 3:**
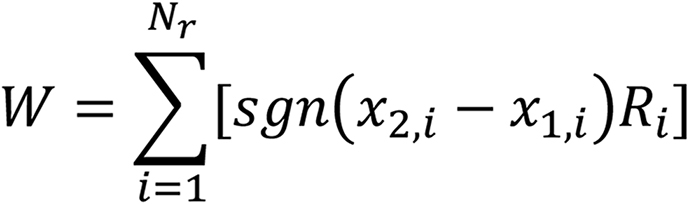
Wilcoxon test equation (W = test value; Nr = reduced sample number; Sgn = signal function; x1 and x2 = values to be compared; R = rank of values).

**Figure 4:**
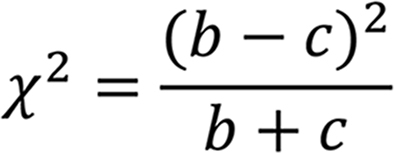
McNemar test equation ($\chi^{2}$ = test statistic, follows chi-square distribution, with one degree of freedom; b = value of cell b in the contingency table; c = value of cell c in the contingency table).

**Figure 5:**
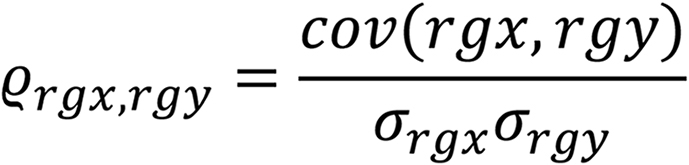
Spearman Correlation test equation (ϱ = Spearman correlation coefficient; *cov(rgx,rgy)* = covariance of variables in ranks; σ_*rgx σ_rgy* = standard deviations of variables in ranks).

The data previously obtained with the help of the Microsoft® Excel 2016 program was organized to elaborate the database, and later the SPSS (Statistical Package for Social Research) version 21.0 program was used for statistical analysis. Data distribution was verified using the Kolmogorov-Smirnov test as a function of sample size. To evaluate the differences between proportions, the tests were used: Chi-squared test, Wilcoxon and Mc Nemar. The equations and models for the methods are shown in Figures 2, 3, 4, and 5. The Spearman Rank Correlation Coefficient test was used to analyze strength of the linear relationship. The level of significance of 0.05 was set for this longitudinal study.

## 3 Results

After the analysis of the inclusion and exclusion criteria, the sample consisted of 163 participants, with a median age of 20 years. There was a predominance among female participants (74%) and singles (93%). Only 7% (11) were married or had a stable union.

Regarding previous diseases, 93% (152) had no associated pathology and only 2% (3) had asthma and the presence of students with heart problems (1%) and Attention Deficit Hyperactivity Disorder (ADHD) (1%). In addition, when asked if they suffered from headaches, 60% (97) of the participants reported that they did not (see Table 1).

**Table 1:** Social and clinical characterization of the sample.

	Median	Percentile 25th	Percentile 75
**Age (years)**	20	19	22

**Sex**			

Female	74% (120)		
Male	26% (43)		

**Marital status**			

Single	93% (151)		
Married	7% (11)		
Widowed	1% (1)		
**Curse**			

Physical therapy	32% (52)		
Dentistry	36% (59)		
Nurse	13% (21)		
Nutritionist	10% (16)		
Physical educator	1% (1)		
Pharmaceutical	4% (7)		
Engineer	4% (7)		
**Previous disease**			

No	93% (152)		
Yes	7% (11)		
**Which previous disease?**			

Asthma	2% (3)		
Heart Problem	1% (2)		
Hypertension and Asthma	1% (1)		
Anxiety	1% (1)		
**Headache**			

No	60% (97)		
Yes	40% (66)		

During the pandemic, social isolation was established in order to reduce coronavirus transmission, in view of this 84% (137) of the interviewees reported being in social isolation and 89% (145) of them claim to be isolated only with their parents.

When the comparison was made between the initial moment and the final moment of collection, there was a significant difference in the weekly time spent in physical activities (p < 0.001) and in seven regions evaluated by the NMQ (cervical; shoulders; arms; wrist, hands and fingers; dorsal, lumbar, hip and lower limbs regions - p < 0.001) (Table 2).

**Table 2:** Comparison between PSQI, IPAQ, MIDAS, NMQ before the pandemic with the final moment of the study.

		Initial	Final	p-value
		% (n)	% (n)	
PSQI				
	Bad sleeper	84% (136)	75% (122)	0.261^a^
	Good sleeper	17% (27)	25% (41)	

IPAQ				
	Sedentary	34% (55)	61% (99)	<0.001^b^
	Active	66% (108)	39% (64)	

MIDAS				
	No Disability or Minimal	59% (96)	60% (98)	0.939^a^
	Mild Disability	17% (28)	13% (22)	
	Moderate Disability	13% (22)	18% (29)	
	Severe Disability	10% (17)	9% (14)	

NMQ				
Cervical spine	No	21% (34)	42% (69)	<0.001^a^
	Some times	39% (64)	23% (38)	
	Frequently	33% (53)	25% (40)	
	Always	7% (12)	10% (16)	

shoulders	No	48% (78)	64% (105)	<0.001^a^
	Some times	29% (48)	17% (28)	
	Frequently	18% (29)	13% (21)	
	Always	5% (8)	6% (9)	

Arms	No	44% (72)	70% (114)	<0.001^a^
	Some times	37% (60)	17% (28)	
	Frequently	17% (27)	10% (17)	
	Always	2% (4)	2% (4)	

Elbows	No	75% (122)	87% (141)	0.001^a^
	Some times	19% (31)	10% (17)	
	Frequently	6% (9)	3% (5)	
	Always	1% (1)		

forearms	No	57% (93)	69% (112)	0.001^a^
	Some times	28% (45)	22% (36)	
	Frequently	12% (20)	8% (13)	
	Always	3% (5)	1% (2)	

fist/hands and fingers	No	21% (35)	40% (65)	<0.001^a^
	Some times	40% (65)	29% (47)	
	Frequently	26% (43)	22% (36)	
	Always	12% (20)	9% (15)	

Dorsal spine	No	52% (85)	69% (112)	<0.001^a^
	Some times	23% (38)	11% (18)	
	Frequently	17% (27)	15% (25)	
	Always	8% (13)	5% (8)	

Lumbar spine	No	69% (113)	55% (89)	<0.001^a^
	Some times	25% (41)	18% (30)	
	Frequently	4% (7)	17% (28)	
	Always	1% (2)	10% (16)	

Hips/legs	No	41% (67)	79% (128)	<0.001^a^
	Some times	25% (40)	13% (22)	
	Frequently	20% (33)	7% (12)	
	Always	14% (23)	1% (1)	

a Wilcoxon test.

b Mc Nemar test; Pittsburgh Sleep Quality Index (PSQI); International Physical Activity Questionnaire (IPAQ); Migraine Disability Assessment (MIDAS); Nordic Musculoskeletal Questionnaire (NMQ), Lower Limbs (LLLl).

When we evaluated the association between the research data, it was observed that there was no statistical difference between the time and quality of sleep and the time spent weekly in physical activities, because it was found that at the beginning of social isolation 84% (47) were sedentary and had the presence of sleep disorder or considered poor sleep, while 83% (89) of them were active, practiced physical activity regularly, however, they also had poor sleep or sleep disorder (p = 0,663). Three months later, it was observed that there was no difference in the association of data, because 88% (77) of the participants started to consider themselves sedentary and with the presence of sleep or poor sleep disorders and among the active, 70% (45) had the same complaints (p = 0.557) (Table 3).

**Table 3:** Association between data from the Pittsburgh Sleep Quality Index and the International Physical Activity Questionnaire.

	IPAQ INITIAL		p-value
PSQI Initial classification	Sedentary % (n)	Active % (n)	
Bad sleeper	84% (47)	83% (89)	
Good sleeper	15% (8)	17% (19)	

	**IPAQ FINAL**		**p-value**
PSQI Final classification	**Sedentary % (n)**	**Active % (n)**	
Bad sleeper	78% (77)	70% (45)	
Good sleeper	22% (22)	30% (19)	

^a^Test of Chi Square; Pittsburgh Sleep Quality Index (PSQI); International Physical Activity Questionnaire (IPAQ).

Table 4 shows results of the association between time, sleep quality (PSQI) and the presence of migraine (MIDAS), there was a statistically significant difference at the beginning of the pandemic (p < 0.001) and at the end of data collection, three months after the social isolation started (p = 0.002). It can be observed that at the beginning of social isolation, 50% (11) participants had moderate migraine and sleep disorder and another 59% (10) had severe disability due to migraine associated with poor sleep quality. Three months after the beginning of isolation, there was a change in relation to these values, because it was found that 50% (7) reported severe disability due to migraine associated with the presence of sleep disorder. In addition, 69% (20) reported having moderate migraine associated with poor sleep quality.

**Table 4:** Association between migraine disability assessment and Pittsburgh sleep quality index data.

	MIDAS INITIAL				p-value
PSQI Initial Classification	Little or no disability % (n)	Mild disability % (n)	Moderate disability % (n)	Severe disability % (n)	
Bad sleeper	79% (76)	86% (24)	95% (21)	88% (15)	<0.001^a^
Good sleeper	21% (20)	14% (4)	5% (1)	12% (2)	
	**MIDAS FINAL**				
PSQI Final Classification	**Little or no disability % (n)**	**Mild disability % (n)**	**Moderate disability % (n)**	**Severe disability % (n)**	
Bad sleeper	67% (65)	86% (19)	90% (26)	86% (12)	0.002^a^
Good sleeper	33% (33)	14% (3)	10% (3)	14% (2)	

a Test of Chi Square; Pittsburgh Sleep Quality Index (PSQI); Migraine Disability Assessment (MIDAS). Disability (Incap.).

Among those who had good sleep quality, 21% (20) reported no migraine related disability at the beginning of the pandemic and three months later, this value increased to 33% (33).

The result of the correlation at the beginning of the pandemic, it was observed where a direct relationship between the Pittsburgh questionnaire points and the number of days with pain (p < 0.001), the Initial MIDAS score (p < 0.001) and the intensity of initial pain (p < 0.001) were observed. The correlation also showed a direct relationship between the intensity of the initial pain and the Initial MIDAS Score (p < 0.001).

The analysis of the correlation at the final moment presented in Table 5 showed a direct relationship between pittsburgh questionnaire points at the end of the study and age (p = 0.044), the MIDAS score (p < 0.001) and pain intensity at the end of the study (p < 0.001). We also observed a direct relationship between the number of days with pain and the Pittsburgh questionnaire points at the end of the study (p < 0.001) and pain intensity (p = 0.003). We also identified a direct relationship between the MIDAS score with the number of days with pain (p < 0.001) and the intensity of pain (p < 0.001) at the end of the study.

**Table 5:** Association between age, Pittsburgh Sleep Quality Index, Migraine Disability Assessment, number of days and pain intensity at the end of the pandemic.

		Age	Points PSQI	Number of pain days	score MIDAS
Points PSQI					
	Correlation coefficient	0.158			
	p-value	0.044			
Number of pain days					
	Correlation coefficient		0.282		
	p-value		<0.001		
Score MIDAS					
	Correlation coefficient		0.369	0.616	
	p-value		<0.001	<0.001	
Pain initial intensity					
	Correlation coefficient		0.304	0.23	0.287
	p-value		<0.001	0.003	<0.001

∗Spearman Rank Correlation Coefficient; Pittsburgh Sleep Quality Index (PSQI); Migraine Disability Assessment (MIDAS).

## 4 Discussion

During this study, there was a predominance in female population and most students were single, which may be related to the low age group. These results are consistent with those found in the study by Regiani et al [16], since 62,84% participants were female and a little more than half were age group between 18 and 20 years (50.19%). It is verified that women are more accessible and participative in studies, which may refer to the number of health courses that participated in data collection, which is known to predominate female students.

Regarding musculoskeletal pain, it was observed that there was a significant difference in seven regions evaluated by the NMQ (p < 0.001), when compared to the data between the initial and final moment of collection, presenting an increase in painful symptoms in several regions, especially in the cervical region, shoulders and arms; being in line with the data presented in some studies [5], whose results showed mainly that 55.8% felt pain in their neck, 54.8% felt pain in their shoulders. It can be assumed in our study that social isolation, during the COVID-19 pandemic, probably caused a significant difference in the weekly time spent in physical activities (p < 0.001) and in seven regions evaluated by the NMQ (p < 0.001).

Also regarding these data, another study obtained results consistent with those found in this paper, because it presented a higher prevalence of musculoskeletal pain in the neck (32.50%), shoulder 26.91%, upper back 20.69%, wrist and hand 19.75% [17]. In another study by the same author, the results of the Nordic Questionnaire showed that musculoskeletal pain were higher in the neck (90.00%), followed by shoulders 73.30% and upper back 63.30% [18]. Such results may be related to the wrongly adopted posture in the use of devices (smartphones, tablets, notebooks) to access classes during the pandemic.

About the presence of sleep disorders associated with physical activity, there was no statistical difference in comparison of the research data, because the vast majority already had some sleep disorder at beginning of the pandemic and also considered themselves sedentary (84%). These data are in line with other research that found that almost 70% of patients reported at least one difficulty in falling asleep, frequent awakenings, non-restorative sleep or waking up too early associated with the home confinement caused by the pandemic [19]. In our research, there was a significant difference in the association between time, sleep quality (PSQI) and the presence of migraine (MIDAS), at the beginning of the pandemic (p < 0.001) and at the end of data collection, three months after starting social isolation (p = 0.002). A direct relationship was also observed between the Pittsburgh questionnaire points and the number of days with pain (p < 0.001), the Initial MIDAS score (p < 0.001) and the initial pain intensity (p < 0.001) in the correlation made between the data at the beginning of the pandemic, in addition to showing a direct relationship between the intensity of the initial pain and the Initial MIDAS Score (p < 0.001).

College students tend to have a greater predisposition to the emergence of sleep disorders, due to the exhausting and stressful routine; because in addition to the curricular activities, many of the students also complement their training by participating in courses, internships, scientific initiation programs, monitoring, extension projects, requiring greater availability of time to carry out all these activities, leading many not maintain adequate quality and/or amount of sleep. At the end of data collection, a direct relationship was observed between Pittsburgh questionnaire points and age (p = 0.044), the MIDAS score (p < .001) and pain intensity (p < 0.001). We also observed a direct relationship between the number of days with pain and the Pittsburgh questionnaire scores (p < 0.001) and pain intensity (p = 0.003). We also identified a direct relationship between the MIDAS score and the number of days with pain (p < 0.001) and pain intensity (p < 0.001) at the end of the study.

It is observed that with social isolation the frequency of physical activity (PA) suffers a fall, in another study the authors report that before the distancing 67.8% of the children performed PA at least twice a week and after the restrictions imposed, 46.1% of the parents reported that the children are practicing much less PA than during the school period, in addition they also consider that there was a 38% increase in the use of screen time [20].

In another study, the percentage levels of PA during social isolation were analyzed and it was found that 59.2% of the participants became sedentary during the pandemic, thus confirming the increase in inactivity from 19% to 36.7%. Despite this reduction, it was found that activities such as walking and running continued to be the most practiced, although in a reduced way. Thus, there was a significant reduction in the practice of exercises in participants who were physically active and very active before the pandemic [21].

The increase in sedentary lifestyle caused by pandemic is easily explained by the fact that spaces used for physical activity (gyms, sports clubs) have been closed due to protocols established by the government in order to minimize the spread of COVID-19 and practice PA at home is often discouraging. Ahmed et al [22] showed the lockdown has led to the various changes in the overall activities and lifestyle and the reduction of physical activity level had profoundly negative impact on musculoskeletal health. Therefore, social isolation, an important measure used by governments to inhibit the spread of SARS-COV-2, has further increased the rates of sedentary lifestyle, which is an aggravating factor for COVID-19, as its negative effects lead to increased stress, anxiety, depression and even immune depression [23]. Our data show the probable potentiation of painful symptoms in different parts of the body, worsening sleep quality and migraine and a decrease in physical activity. There seems to be an association between sleep quality and the number of days with pain and its intensity with migraine. There also seems to be a relationship between age and sleep quality among young people.

This study has some public health implications increasing public health strategies and policies. Regarding migraine associated with sleep disorders, no significant improvements were observed, and such results were inconsistent with those found in the study that says that there was a general improvement in migraine in the population studied due to the reduction of stress related to school activities during social isolation, 46% reported that there was improvement and only 15% presented worsening in pain [24]. However, in another study, it was found that 47% of the participants reported a reduction in sleep quality during isolation. Subjectively, the main factors associated with this reduction would be the change in sleep behavior and reduced exposure to daylight and increased use of screens at night [25]. Health can be linked to public policies and the integration of health with adequate practices of healthy habits policies can emphasize changing lifestyles.

There are limitations in the study design and sampling method due to the type of study, self-reported findings may provide more desirable answers. The lack of representation of the entire population affects external validity, despite the measures described in the method to identify possible biases, research of this nature can select individuals inappropriately (it is possible that the most socially engaged people participate of the study). The fact that it is a longitudinal study brought difficulties and limitations for its execution, because due to the fall in the number of participants between the first (n = 363) and the second phase (initial n = 197) of the collection, we chose not to insist on the students to participate again in the research, thus limiting the number of participants, and perhaps the results of the findings.

## 5 Conclusion

Social isolation, during the COVID-19 pandemic, probably potentiated painful symptoms in various parts of the body, worsening sleep quality and migraine. In addition, there is strong evidence that the decrease in physical activity during the pandemic is associated with sleep quality, with the number of days, musculoskeletal pain and migraine. Musculoskeletal pain, poor sleep quality and migraine can become a long-term problem, among this fact, we suggest initiatives to provide social and emotional support to young people that encourage the habit of physical activities and adequate interventions should be planned to improve the health condition of this population. It seems that the disorders mentioned throughout the research can be alleviated through the practice of physical activity and the fight against sedentary lifestyle, as physical exercises are an important component of a healthy lifestyle and bring several health benefits. Finally, the COVID-19 pandemic may be associated with biological and social interactions that can worsen the quality of people’s health if there is not adequate interference. Future studies can be done to associate the long-term effects of the pandemic on health and provide new directions for research and treatment of these symptoms, in addition to guiding public policies for integrating health with changes in lifestyles.

## Declarations

### Author contribution statement

Juliana Pedrosa Luna Oliveira: Conceived and designed the experiments; Contributed reagents, materials, analysis tools or data; Analyzed and interpreted the data; Wrote the paper.

Juliana Zangirolami-Raimundo: Contributed reagents, materials, analysis tools or data; Analyzed and interpreted the data; Wrote the paper.

Paulo Evaristo de Andrade, Luiz Carlos de Abreu: Conceived and designed the experiments; Wrote the paper.

Soraya Louise Pereira Lima, Amanda Regina Cavalcante Lima: Contributed reagents, materials, analysis tools or data; Wrote the paper.

Rodrigo Daminello Raimundo: Conceived and designed the experiments; Contributed reagents, materials, analysis tools or data; Wrote the paper.

### Funding statement

This research did not receive any specific grant from funding agencies in the public, commercial, or not-for-profit sectors.

### Data availability statement

Data will be made available on request.

### Declaration of interest’s statement

The authors declare no conflict of interest.

### Additional information

No additional information is available for this paper.
